# Assessment of Otolaryngology Residency Training Program in Iran: Perspectives of Faculty Members and Recently Graduated Medical Students

**Published:** 2019-01

**Authors:** Mohammad Faramarzi, Mohammad Hossein Mohammad Hossein, Mitra Amini, Sayed Taghi Heydari, Azadeh Samiei, Masoud Motasaddi Zarandy, Ali Eftekhari, Mohammad Mahdi Ghasemi, Mohammad Hossein Baradaranfar, Masoud Naderpour, Ajalloueyan Mohammad, Sulmaz Mohammadi

**Affiliations:** 1 *Department of Otolaryngology, School of Medicine, Shiraz University of Medical Sciences, Shiraz, Iran.*; 2 *Research Center for Health Sciences, Institute of Health, Shiraz University of Medical Sciences, Shiraz, Iran.*; 3 *Clinical Education Research Center, Shiraz University of Medical Sciences, Shiraz, Iran. *; 4 *Health Policy Research Center, Institute of Health, Shiraz University of Medical Sciences, Shiraz, Iran. *; 5 *School of Medicine, Shiraz University of Medical Sciences, Shiraz, Iran. *; 6 *Otolaryngology Research Center, Shahid Sadoughi University of Medical Sciences. Yazd, Iran.*; 7 *School of Medicine, Tabriz University of Medical Sciences, Tabriz, Iran.*; 8 *School of Medicine, Baqiyatallah Medical Sciences University, Tehran, Iran. *

**Keywords:** Education, Faculty, Graduate, Otolaryngology, Otology

## Abstract

**Introduction:**

There is limited evidence regarding the quality of otolaryngology residency programs in Iran. Regarding this, the present study aimed to assess some aspects of otolaryngology residency program in the field of otology in Iran based on the perspectives of faculty members and graduates.

**Materials and Methods:**

This study was conducted on 105 recent graduates and 30 faculty members and/or program directors in otolaryngology using two self-administered questionnaires.

**Results:**

While the faculty members believed that a resident should work on at least 5.4 temporal bone surgeries on average, the actual number was 2.49. Tympanoplasty was assigned the highest rate of satisfaction by the recent graduates, whereas the lowest score belonged to middle ear exploration, ossiculoplasty, and stapes surgery. Only 53.6% of the graduates stated that there was an organized training curriculum in temporal laboratory. The recent graduates reported to have more frequent experiences of performing usual otology operations. However, they had fewer experiences of performing more advanced surgeries. The recently graduated subjects had a significantly low level of satisfaction with their competencies in carrying out more complex types of otology surgeries.

**Conclusion:**

High prevalence of otology surgeries in Iran provides valuable opportunities for training otolaryngology residents to achieve an acceptable level of competency. However, the results of this study strongly suggest the necessity of quality improvement both in teaching-learning and assessment processes in otolaryngology training programs.

## Introduction

Empowerment and preparation of medical students for the delivery of effective health care in a safe and acceptable manner is a major aim of medical education (1,2). However, many studies have shown that a significant proportion of medical graduates do not achieve necessary preparedness for carrying out their professional task competently (1,2). Preparedness depends mainly upon the extent to which a trainee has had first-hand experiences during his/her academic years. 

Therefore, clinical education and hands-on training require adequate exposure to various cases at workplace (3). Nonetheless, this end may not be attainable due to such factors, as priority of patient safety, competitiveness between trainees, or rarity of cases (1). In addition, some of the other important factors affecting this issue include high patient’s expectations, patients' awareness of their rights, patients’ concerns about the competencies of their physicians, shortened hospital stay, and balancing efficiency with education (4,5).

Medical residency programs, such as ophthalmology and family medicine (6), have been evaluated in different studies. Specifically, some studies have focused on exploring the surgical competencies of otolaryngology residents. In a study conducted by Bath and Wilson in the UK, it was stated that there was a noticeable improvement in surgical ability and competencies of residents at the end of their training (7). 

In a similar study performed by Georgalas et al., otolaryngology training in the UK was reported to be unified, successful, and satisfactory (8). On the contrary, in a survey study conducted by Carr in the United States, an inconsistency was found between the opinions of program directors and graduates about the number of the procedures required to achieve competency (9). Similarly, Komiya et al. reported that Japanese trainees in otolaryngology subspecialty did not achieve optimal mastery in surgical techniques during their post-residency period (10). 

Some studies show that assessments, such as national board exams, do not reflect the whole reality about the quality of the training programs and the graduates’ qualifications (11). In a study, Geoffrion et al. evaluated surgical skill assessment methods in nine surgical specialties during residency program in all 17 Canadian medical schools. They indicated that subjective evaluation by faculties was the most common method of competency assessment at graduation in all surgical specialties (12). 

In addition, many experts believe that the implementation of a comprehensive participatory evaluation approach involving the main stakeholders, such as program directors and trainees, is necessary to obtain a proper view of the quality of the programs. For this purpose, various methods are available for evaluating the different components of training programs in medical education (13). Similar to the educational programs of other countries, otolaryngology residency program in Iran takes 4 years (10). This program is executed in 13 training centers in the country. The dominant form of education in residency program is apprenticeship model. The training schedules may vary from country to country , because the acquisition of professional competency is context-dependent (14). With this background in mind, the present study was conducted to evaluate the efficacy of training in common otology surgeries during residency program from the perspectives of faculty members and recently graduated medical students. 

## Materials and Methods

This multi-center, cross-sectional study was conducted on otolaryngology training centers throughout Iran under the supervision of Shiraz University of Medical Sciences, Shiraz, Iran, from Jun 2016 to March 2017. Out of the total departments of otolaryngology of 10 medical universities affiliated to Iran Ministry of Health and Medical Education, nearly 30 faculty members and/or program directors and 105 recently graduated otolaryngology students were identified and contacted through phone calls and/or emails. Two separate self-administered questionnaires were sent to the participants by email after receiving their consent for participating in the study. The validity of the questionnaires were confirmed by a panel of experts (n=5) in otolaryngology, rendering a content validity index of 0.89 (none of the items was less than 0.78) and a content validity ratio of 1 for all items, except for one (0.60). The main items of the questionnaire targeting graduates included concerns about the existence of an equipped temporal bone laboratory in the training center and a planned and compulsory training program in temporal laboratory. The participants were asked to specify their level of experience in each type of otology surgeries and satisfaction level regarding their competency in common otology surgeries after graduation. There were some similarities between the faculty members and graduates' questionnaires. However, the questionnaires addressing faculty members entailed some items to inquire the ideas of faculty members regarding the number of temporal bone surgeries that a resident had to perform to acquire adequate competency to be allowed to independently carry out these procedures in an operating room. They were also asked to estimate the ideal number of operations that should be observed, assisted, and performed with a supervisor attendance and also independently during the residency period.

All potential respondents were informed about the objectives of the study and voluntariness of participating in the study. With regard to the cross-sectional design of the study and data collection method (i.e., via emails), respondents’ consent for participating in the study was ensured by their reply to the emails containing the complete questionnaire. It is also worth noting that confidentiality was respected in this study. The data were analyzed in SPSS software (version 11.5) using descriptive and inferential statistics (e.g., Chi-square test). P-value less than 0.05 was considered statistically significant. 

**Ethical considerations**

All procedures performed in this study involving human participants were in accordance with the ethical standards of the Research Council and Ethics Committee of Shiraz University of Medical Sciences, Shiraz, Iran (code: 1786).

## Results

A total of 25 faculty members and/or program directors (response rate: 83%) and 69 graduates (response rate: 66%) answered and returned the questionnaires. Based on the results, 85% of the graduates mentioned that their training center had an equipped temporal bone laboratory, and 53.6% of them stated that there was an organized training curriculum in temporal laboratory. Approximately, 66.7% of the graduates indicated that "this curriculum was a compulsory program".

On the other hand, 84% of the faculty members believed that working on temporal bone is obligatory. In addition, all faculty members agreed that working on temporal bone before entering the operation room should be obligatory. Furthermore, 97.1% of the graduates reported that they had worked on temporal bone. In this regard, the mean number of the temporal bone dissections that they had performed during their residency was 2.49. However, faculty members believed that a resident has to work on at least a mean number of 5.4 temporal bone dissections.

The faculty members were also asked about the number of operations needed to be performed by residents on temporal bone before entering the operation room. In the same vein, the graduates’ were required to self-report the actual number of temporal bone surgeries that they had performed for each of six operations in residency. As Table 1 indicates, ossiculoplasty, stapes surgeries, middle ear explorations (MEE), and tympanoplasty were the four operations that had been carried out less frequently than the required mean number stated by the faculty members. 

**Table 1:** Comparison of the required mean number of temporal bone operations according to faculty members’ opinion and the mean number of actual operations reported by graduates

**Types of otology operations**	**Number of operations needed (faculty members’ opinion)**		**Number of operations performed (graduates’ report)**		**P-value**
	**Mean**	**SD**	**Mean**	**SD**	
Tympanoplasty	4.80	3.93	0.78	2.01	<0.001
Intact canal wall mastoidectomy	5.64	2.43	4.58	4.82	0.298
Canal wall down mastoidectmy	5.68	2.38	4.13	4.37	0.097
Middle ear exploration	5.88	4.14	2.48	2.77	<0.001
Ossiculoplasty	6.20	3.66	0.49	1.09	<0.001
Stapes surgery	7.08	6.32	0.43	0.93	<0.001

Graduates reported frequent experiences of performing routine otology operations, either independently or with minor help. They performed about 35 tympanoplasties and 31 intact canal wall mastoidectomies (ICWM). However, they had fewer independent experiences in more advanced operations, such as MEE (n=6), ossiculoplasty (n=4), and stapes surgeries (n=1) during their post-residency training period (Table.2). 

**Table 2:** Mean number of common otology surgeries that graduates observed and performed with major help, minor help, and independently

**Types of otology operations**	**Graduates’ levels of experience**			
	**Observed**	**Performed with major help**	**Performed with minor help**	**Performed independently**
Tympanoplasty	25.41±17.47	20.29±15.30	16.57±13.02	35.10±25.65
Intact canal wall mastoidectomy	20.90±11.45	18.49±10.87	18.46±19.63	31.46±23.81
Canal wall down mastoidectomy	15.58±11.49	12.80±10.06	10.56±8.47	15.17±12.21
Middle ear exploration	13.70±8.31	9.55±8.88	7.09±7.59	6.00±8.96
Ossiculoplasty	9.97±6.61	6.33±5.24	3.59±3.79	4.03±6.08
Stapes surgery	9.61±9.73	3.99±4.33	1.80±2.66	1.52±3.38

Graduates reported a higher level of satisfaction with their competency in performing tympanoplasty, ICWM, and canal wall down mastoidectomy (CWDM). However, they had a significantly low satisfaction mean score regarding their competency in carrying out more complex types of otology surgeries, such as MEE, ossiculoplasty, and stapes surgeries (Table.3). 

**Table 3:** Mean scores of graduates’ self-rated satisfaction with their competency in common otology surgeries after graduation

**Types of otology operations**	**Satisfaction score**		
	**Min**	**Max**	**Mean±SD**
Tympanoplasty	0	10	4.96±4.22
Intact canal wall mastoidectomy	0	10	4.29±4.25
Canal wall down mastoidectomy	0	10	3.25±4.05
Middle ear exploration	0	10	1.48±3.08
Ossiculoplasty	0	10	1.62±3.11
Stapes surgery	0	10	0.43±1.70

A comparison was made between the opinions of the faculty members and graduates about the desired number of otology operations, which are needed to be observed and performed with assistance and independently for acquiring acceptable competency levels during residency period (Table 4). The mean scores for the operations to be observed ranged from 14.52±9.52 for MEE to 21.65±19.69 for ICWM. In comparison with the faculty members, the graduates believed that a greater number of MEE and stapes surgeries are needed to be observed (P<0.001); however, the opinions were reversed in terms of ossiculoplasty (P<0.001). The opinions of the faculty members and graduates were similar, without any statistically significant differences, regarding the mean scores of otology operations needed to be observed, assisted, or performed independently during the residency training period. 

**Table 4:** Comparison of faculty members and graduates’ perspectives concerning the ideal number of otology operations residents need to experience during residency training period

**Chronic otitis media ** **surgeries**		**Opinions**	**Observed**					**Assisted **					**Performed independently**				
			**Mean**			**SD**		**Mean**	**SD**				**Mean**	**SD**			
Tympanoplasty		Faculty	15.84			8.285		17.36	11.60				22.60	14.73			
		Graduates	19.62			15.09		17.88	14.65				25.17	20.57			
P-value			0.238					0.873					0.575				
Intact canal wall mastoidectomy		Faculty	15.92			8.91		17.32	11.29				22.60	13.93			
		Graduates	21.65			19.69		20.99	19.36				26.4	20.22			
P-value			0.170					0.368					0.244				
Canal wall down Mastoidectomy		Faculty	16.96			11.058		17.00	11.37				22.40	14.15			
		Graduates	18.55			14.55		17.42	13.75				22.07	15.53			
P-value			0.617					0.888					0.928				
Middle ear exploration		Faculty	14.52		9.56			14.52		1018			18.40		12.29		
		Graduates	16.58		9.85			14.93		9.89			17.99		12.47		
P-value			0.001					0.991					0.601				
Ossiculoplasty		Faculty	15.64	8.45				16.44			9.82		19.56			12.39	
		Graduates	11.72	11.11				15.59			10.13		20.65			18.62	
P-value			<0.001					0.181					0.306				
Stapes surgery	Faculty		15.52				8.21	15.40				8.45	17.62				11.68
	Graduates		17.36				11.84	15.22				9.87	19.39				18.59
P-value			0.008					0.762					0.094				

The opinions of both faculty members and graduates were analyzed regarding the proper time of learning and training each of the six operations in residency period versus otology fellowship. All graduates and faculty members believed that tympanoplasty, ICWM, and CWDM should be learned during the residency period. About 16% of the faculty members believed that MEE must be learned during the otology fellowship; however, this rate was only 1.4% in the graduates. In addition, 48% of the faculty members and 24.6% of the graduates indicated the fellowship program as the appropriate time for training/learning ossiculoplasty. Furthermore, 84% of the faculty members and 52.2% of the graduates preferred the fellowship as an appropriate time for training/learning the stapes surgeries. 

## Discussion

In this study, some clear evidence was presented to show the strengths and weaknesses of common otology surgery training in Iran. Faculty members/program directors and recently graduated students were two important key stakeholders participated in the present study. Actually the mean number of operations performed on temporal bone by residents was lower than that assumed to be proper by the faculty members, especially regarding MEE, ossiculoplasty, and stapes surgery.

 In addition, tympanoplasty obtained the highest graduates’ satisfaction score whereas the lowest scores in this regard were assigned to CWDM, MEE, ossiculoplasty, and stapes surgery. In the same vein, tympanoplasty was the most observed and performed surgery, whereas MEE, ossiculoplasty, and stapes surgery were the least observed and performed operations. As shown in Table 2, the graduates believed that they had been subjected to sufficient number of chronic otitis media (COM) surgeries, such as tympanoplasty, IDWM, and CWDM, during training; however, they were seemingly disappointed with the outcome in their postgraduate period (Table.3). Based on the evidence, proficiency is not associated with the quantity of operations performed during training (15-17). It seems that the logical and practical solution is to deviate away from merely counting case numbers and move toward the objective evaluation of procedural competence (18). 

Other researchers have also investigated the educational status of their country. In a hospital-based cross-sectional study performed by Taleb Abadel and Saeed Hattab, it was found that junior physicians tend to overestimate their own clinical abilities. Accordingly, they observed a wide gap between the graduates' self-assessment and faculty members' evaluation regarding their abilities (11). Similarly, in a study conducted by Keith A et al., residents rated their competency much higher than expected by faculty members in most of the procedures, such as tympanoplasty without mastoidectomy, tympanoplasty without ossicular chain reconstruction, simple mastoidectomy, and stapedectomy (18).

On the other hand, in a study conducted by Cave et al. in the UK, 42% of the new postgraduates of medical schools felt that they had not acquired sufficient level of competency to start practicing (19). In addition, in a study performed by Peyre et al., residents evaluated their own laparoscopic skills lower than that expected by the faculty members (20). Bath and Wilson demonstrated that otolaryngology trainees were gradually able to perform nearly almost all the procedures independently after 4 years of training. However, this development was slower for some major procedures, such as modified radical mastoidectomy, and they achieved competency during the 5^th^ or 6^th^ years of their training (7). 

In a national survey conducted by Lee et al. on general surgery (GS) and otolaryngology (OTO) in the United State, GS and OTO residents believed that mean numbers of 13 and 25 thyroidectomies, deemed necessary for graduation by their respective review boards, were not sufficient for achieving the desired competency in thyroid surgery. Interestingly, their study showed that there was an evident discrepancy between the number of procedures required for board certification and the number of cases residents believed to be necessary (21). It is remarkable that in the present study, the new graduates had performed approximately 31 unsupervised ICWM during their residency program, and about 85% of them could perform this procedure without any supervisions. 

With respect to CWDM, the residents had carried out about 15 unsupervised surgeries, and about 72% of them could perform this procedure unsupervisedly. The results of the study conducted by Georgalas et al. in the UK revealed that the trainees performed averagely seven modified radical or radical mastoidectomies during their residency. However, only 8% of them were able to perform this procedure under no supervision (8). This inconsistency between our findings and those of Georgalas et al. can be explained by the incidence rate of advanced COM in developing countries. It is logical that surgical training should focus on the most common procedures in society (22). In a study by Carr et al., program directors stated that about 9.6 cases of modified radical mastoidectomies were required to achieve competency, which was greater than the number (6.6 cases) reported by graduates (9).

On the contrary, in the present study, the estimated number reported by program directors for the acquisition of competency in ICWM was less than the reported number by graduates (22.6 versus 26.4 cases). However, the numbers were similar for CWDM. On the other hand, the results of the present study showed that approximately half of the centers implemented an organized training curriculum or obligatory program. 

Human temporal bone dissection is a golden standard method in otologic training (23,24). Some authors have suggested a more reliable scale (i.e., task-based valid checklist) to objectively assess the temporal bone laboratories (25). As other options, junior residents can operate on animal temporal bones or preform virtual reality surgeries prior to working on human temporal bones (26, 27). 

The opinions of faculty members/program directors and graduates were compatible about the appropriate time of residency or fellowship periods for teaching/learning the common and less advanced types of otology surgeries, such as tympanoplasty, ICWM, and CWDM. However, they had different perspectives about more advanced surgeries, such as MEE and stapes surgeries. There are so much controversies regarding ‘who should perform stapes surgery?’ As Ruckenstein and Staab (2008) mentioned, The American Board of Otolaryngology, Head and Neck Surgery considers stapedectomy to be within the competency of general otolaryngologists (28). 

In a study conducted by Carr in the United States, it was reported that 8.1% of program directors believed that stapes surgery was beyond the residents’ expertise level (9). In another study, the researchers revealed that 84% of program directors believed that fellowship training was not essential for otology (29). Meanwhile, some believe that stapes surgery can be performed by well-supervised residents (30). In line with pervious results, some researchers think that all steps of the surgery, except for opening the footplate, are basic and should be taught during the residency program (31). On the contrary, some believe that residents are incapable of preforming stapes surgery (32), and that if they carried out such operations, the rate of complications will rise (31). 

Some studies have indicated a drop in the number of otosclerosis cases over the past few decades (33). In a review, Statham and Pensak (2006) reported that the incidence and prevalence of clinical otosclerosis has undergone a decline, while the number of subspecialties in otolaryngology has been increased during the recent decades. They then concluded that infrequent exposure to disease or limited experience during residency training period implies referring the patient to an expert otologic surgeon for achieving optimal care (34). 

Review of the literature revealed discrepancies among findings regarding the number of required surgeries to obtain an optimum outcome. Several studies revealed that approximately 43-80 surgeries are required (35,36); however, in a study carried out by Caughey et al., it was reported that general otolaryngologists annually perform 1-6 stapes surgeries (37). In the United Kingdom, Georgalas et al. found that residents perform merely four stapes surgeries, and 35% of them felt capable of doing the operation without supervision. Regarding the ossicular reconstruction, the residents were subjected to 12 producers, and 63% of them felt proficient to perform the operation under no supervision (8). RucKenstein and Staab emphasized the importance of training adequacy and surgical experience. Furthermore, they concluded that “it would seem more logical to remove stapes surgery from the list of “core competencies” required for the completion of a residency training program in general otolaryngology” (28). 

The results of the current study are consistent with those of the previous studies. In the present study, it was found that the residents had not performed ossiculoplasty or stapes surgery on temporal bone adequately. The number of ossiculoplasty and stapes surgeries carried out by residents during their residency program was only 4 and 1 cases, respectively. 

Almost 50% of the faculty members and 24.6% of the graduates believed that it is necessary to train ossiculoplasty during the otology fellowship. In addition, most of the faculty members and half of the graduates assumed that learning stapes surgery is essential during the fellowship. In a study conducted by McMains and Peel, it was revealed that only a minority of otolaryngology faculty members in the United States had taken a course regarding the theory and practice of medical education (38). This finding is consistent with that of the present study in underscoring that the achievement of teaching competencies is pivotal for all academic staff.

Furthermore, some authors believe that expert performance is attainable through gaining more experience by deliberate practice. This measure is possible by intentionally altering cognitive mechanism to maintain long-term learning and achieve improvement (15). It has been shown that the trainees’ competence increases over time; however, this increase is not steady (39). Moreover, the accomplished levels of experience and competency are directly related to the time devoted to practice (40,41). In addition, some researchers believe that it takes at least five years for a surgeon to reach the plateau phase in his/her learning curve of tympanoplasty type I (42). Therefore, further objective research is required to confirm the results of the present study. The specialist are also suggested to perform more in-depth self-assessments at least 5 years after graduation. 

The findings of the present study underscore the necessity of redesigning residency program by program directors to make it in line with the demands and requirements of the society. It is also essential to adopt more reliable and objective methods for the evaluation of training in other fields of otolaryngology. This credible evidence about postgraduate performance can be interpreted regarding three different aspects, namely social, educational, and assessment issues, in response to the following questions: 1) how can dissatisfaction with outcome affect the safety of patients in society?, 2) is there any need to rethink or define the crucial requirements of otology training?, and 3) is it necessary to try to improve assessment methods for evaluating surgeon’s abilities? 

It is recommended that more objective and formative evaluation methods, such as task-specific checklists, evaluation portfolios from multiple assessments, mini-clinical evaluation exercise, directly observed procedural skills, and mini-peer assessment tool, be implemented to enhance the reliability of evaluating surgical skills (14,18,43). 

**Limitations of the study**

An important strength of this study was the relatively large sample size and high response rate, particularly from specialists, who provided a comprehensive and generalized view of practice in otology in Iran. However, the only major drawback of this research was its subjective nature. No correlation was observed within specialists' satisfaction scores regarding the outcome and the number of operation performed during residency. This apparent lack of correlation can be attributed to the subjective methodology of the current research. 

Moreover, the authors simply relied on self-reports of specialists to determine their satisfaction level which may have resulted in self-selection bias. Meanwhile, inter-personal differences in achieving surgical skills is a fact which may cause unsatisfactory postoperative outcomes (18,44). Another limitation of the study was the limited number of centers for data collection, which was 10 out of 13 medical universities. The last limitation of the study was the failure to evaluate the reliability of the instrument through running a pilot study.

## Conclusion

Relatively high prevalence of COM cases in our society has provided potential, valuable opportunities that could be used in learning and achieving optimal competency in relevant otology surgeries. Nonetheless, it seems that the lack of an organized training curriculum, unavailability or limited use of temporal bone laboratories, and some deficits in objective performance-based assessment in some training centers have decreased the efficiency and effectiveness of otolaryngology training programs. Therefore, it is suggested to direct attention toward quality improvement in educational processes, including planned utilization of temporal bone laboratories in teaching and assessment of students. 

## Conflicts of Interest

The first author (Mohammad Faramarzi) has received research grants from Shiraz University of Medical Sciences, Shiraz, Iran. This grant was paid for the costs of project execution. All authors declared that they have no conflicts of interest.
